# Orthodontic treatment need in a Spanish young adult population

**DOI:** 10.4317/medoral.17722

**Published:** 2012-02-09

**Authors:** Carlos Bellot-Arcís, José M. Montiel-Company, David Manzanera-Pastor, José M. Almerich-Silla

**Affiliations:** 1 Dentistry graduate. Orthodontic Masters student, Faculty of Medicine and Dentistry, University of Valencia (Spain); 2 Post-Doctoral Assistant Professor, Stomatology Department, Faculty of Medicine and Dentistry, University of Valencia (Spain); 3 Adjunct Professor, Stomatology Department, Faculty of Medicine and Dentistry, University of Valencia (Spain); 4 Tenured Lecturer, Stomatology Department, Faculty of Medicine and Dentistry, University of Valencia (Spain)

## Abstract

Objectives: Orthodontic treatment need has often been assessed in child populations, but few studies employing internationally-recognized indices have been conducted in adult or young adult populations. The aim of this study was to determine the orthodontic treatment need of a young adult population in Spain by means of the Dental Aesthetic Index (DAI), the Index of Orthodontic Treatment Need (IOTN) and the need perceived by the patients. Study design: A cross-sectional epidemiological study was conducted in a broad, representative sample of 671 adults aged between 35 and 44 years using health centers in the Valencia Region of Spain, following the recommendations of the World Health Organization (WHO). Results: Orthodontic treatment was required by 31.3% of the sample according to the DAI and 19.2% according to the IOTN (DHC). The orthodontic treatment need perceived by the patients was 21.1%. On relating treatment need to different variables, significant differences in patient perception were encountered by gender, as women perceived a greater need (23.9%) than men (14.4%). Significant differences in previous orthodontic treatment history were found between middle/high (15%) and low (9%) social class and between secondary/tertiary (14%) and primary (3.3%) education. Conclusions: There was no agreement between the treatment need assessed objectively by the indices and that perceived by the patient, or between the indices themselves. The decision to undergo orthodontic treatment can depend on socioeconomic and psychological factors and on values and principles that do not easily lend themselves to objective measurement.

** Key words:**Orthodontics, epidemiology, adult, malocclusion.

## Introduction

Orthodontics has traditionally focused on children and adolescents. Adults are increasingly seeking orthodontic treatment, yet very few studies have been carried out in this age group (1).

Some authors (2) consider that orthodontic treatment need studies should be undertaken in the adult population, which is considered sufficiently mature to form a sound judgment of the importance of dental esthetics for social acceptance and of the impact this can have in daily life. Adults are also more emotionally stable and their concept of dentofacial esthetics is more realistic (3-8).

A number of recognized indices, validated by various international associations, are available to determine treatment need objectively. These include the Dental Aesthetic Index (DAI) and the Index of Orthodontic Treatment Need (IOTN) (9).

For the above reasons, the present study was conducted to determine the orthodontic treatment need of the adult population of the Valencia region of Spain using DAI and IOTN, analyze the diagnostic agreement between these two indices, detect the subjective treatment need and the relation between this and the objective need determined by the indices, and examine the association between the treatment need detected by the DAI and IOTN and socioeconomic status, level of education and gender.

## Material and Methods

A descriptive cross-sectional epidemiological study was conducted in a representative random sample of the adult population aged 35-44 years in the Valencia Region of Spain, following the recommendations of the World Health Organization (WHO) for this type of study.

Systematic sampling was carried out to determine the national health system health centers in which to conduct the examinations. These were ordered according to the registered population information supplied by the regional government health ministry’s Population Information System (Sistema de Información Poblacional – SIP). All the health centers in the Valencia region were included in the sampling process and a total of 74 were selected. The mean number of interviews to be conducted per team, day and health center was estimated at 15. The examinations were carried out in a room in the health center. The instruments used were a WHO type periodontal probe and a #5 plain mouth mirror.

The study was approved by the University of Valencia Faculty of Medicine and Dentistry ethics committee. Prior to examination, the subjects signed an informed consent form.

Those included in the study were patients from 35 to 44 years of age who were visiting the health center for reasons other than buccodental pathology. Patients receiving orthodontic treatment at the time of examination were excluded. Those who had received orthodontic treatment in the past were not excluded. In the data analysis, those with six or more visible teeth absent (incisors, canines or premolars) were also excluded. The final sample numbered 671 (202 men and 469 women) and the precision of the study was 0.03.

The three chosen examiners were calibrated before the study commenced, assessing the agreement between their diagnoses of orthodontic treatment need using DAI and IOTN in study models and those of a benchmark examiner (acting as ‘gold standard’). The kappa values obtained for the three examiners were 0.85, 0.83 and 0.80. A second calibration was then carried out in 15 adult patients aged 35 to 44 years at the University of Valencia Medicine and Dentistry Faculty’s dental clinic, when the three examiners all obtained kappa values of over 0.85.

For this study, all the variables required for calculating the DAI and IOTN (9) were recorded on an examination form. A ques-tionnaire was also administered for the purpose of analyzing the potential demand for orthodontic treatment, the previous orthodontic history, the perception that adults have of their dental esthetics and the importance they place on them, and the orthodontic treatment need perceived by each subject.

To establish their educational level, those with primary or no schooling were classed as ‘primary’ and those with a baccalaureate, vocational training or a university education were classed as ‘secondary/higher’. For the classification of socioeconomic status, the UK Registrar General´s categorization of occupational social class (10) was employed. Classes I - professional, II – managerial/technical and III - skilled non manual and skilled manual were classed as ‘middle/high’. Classes IV - partly skilled manual y V - unskilled manual were considered ‘low’. Others were considered non-classified.

The data collected on the examination forms and questionnaires were stored in a database, using Microsoft® Access 2007® software. Statistical analysis was performed with SPSS v 15.0® software.

The chi square test was used for comparison of proportions at a 0.05 significance level. To determine the diagnostic agreement between indices, the Kappa statistic and the percentage agreement were employed.

## Results

 Table 1 shows the orthodontic treatment need as determined by the DAI and IOTN and perceived by the patient. The mean DAI was 28.3, with a 95% confidence interval of 27.7-28.9. 31.3% of the sample were classed as DAI grades 3 and 4 and were therefore considered to need orthodontic treatment. According to the IOTN DHC results, 19.2% of the adult population in the 35-44 age group would need orthodontic treatment. The need perceived by the patients was 21.1%. On analyzing the agreement between the diagnoses indicated by the DAI and IOTN, this was found to be 71.6%, with a Kappa index score of 0.26. The agreement between DAI and self-perception was 67.3%, with a Kappa of 0.17, and that between IOTN and self-perception was 76.2%, with a Kappa of 0.26.

**Table 1:**
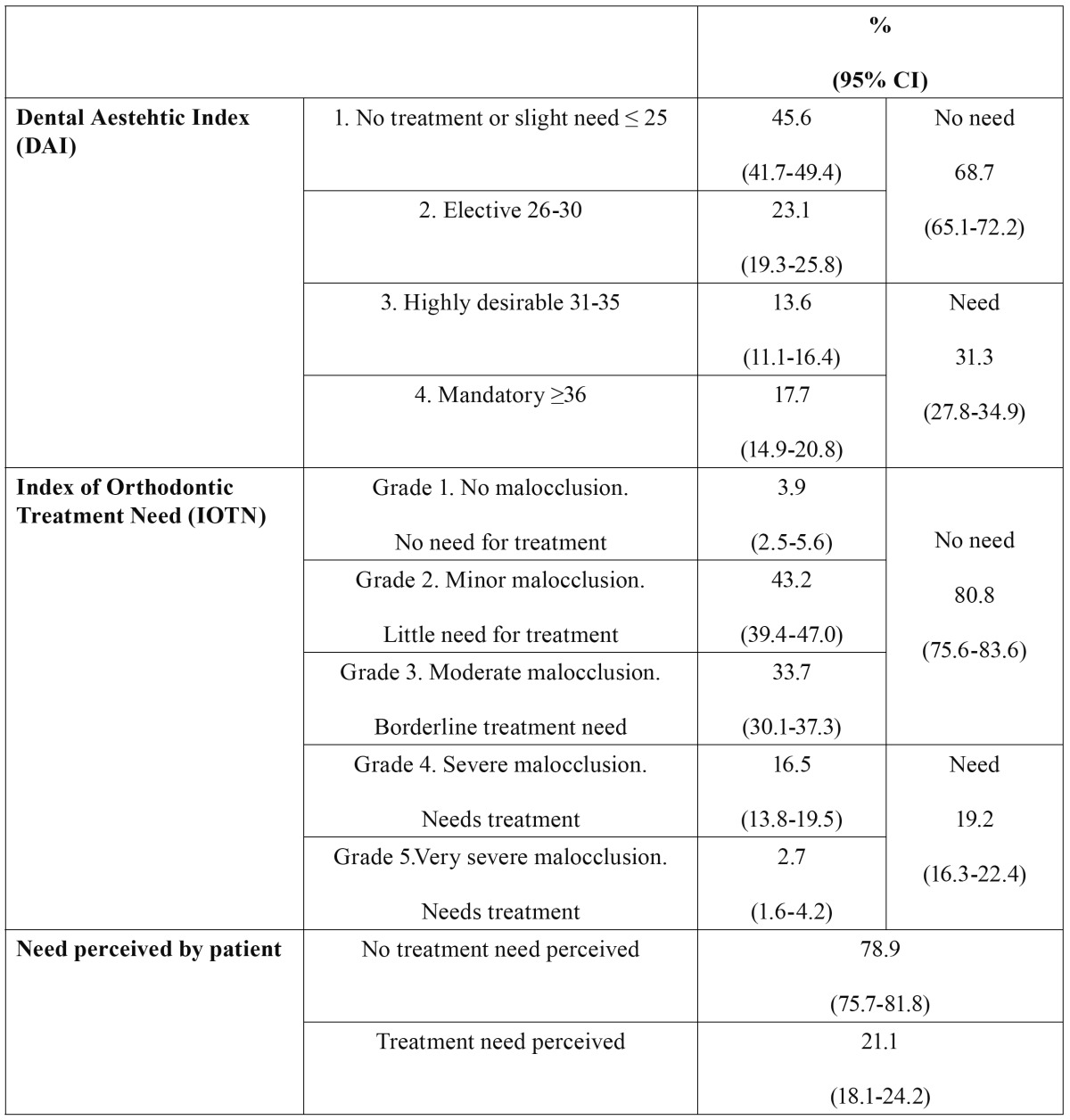
Distribution of treatment need levels as determined by the DAI and IOTN and perceived by patients (n=671).

On relating the treatment need to the variables of gender, socioeconomic status and education ( Table 2), the only significant differences in the patients’ perception were by gender, being higher in women (23.9%) than men (14.4%).

**Table 2:**
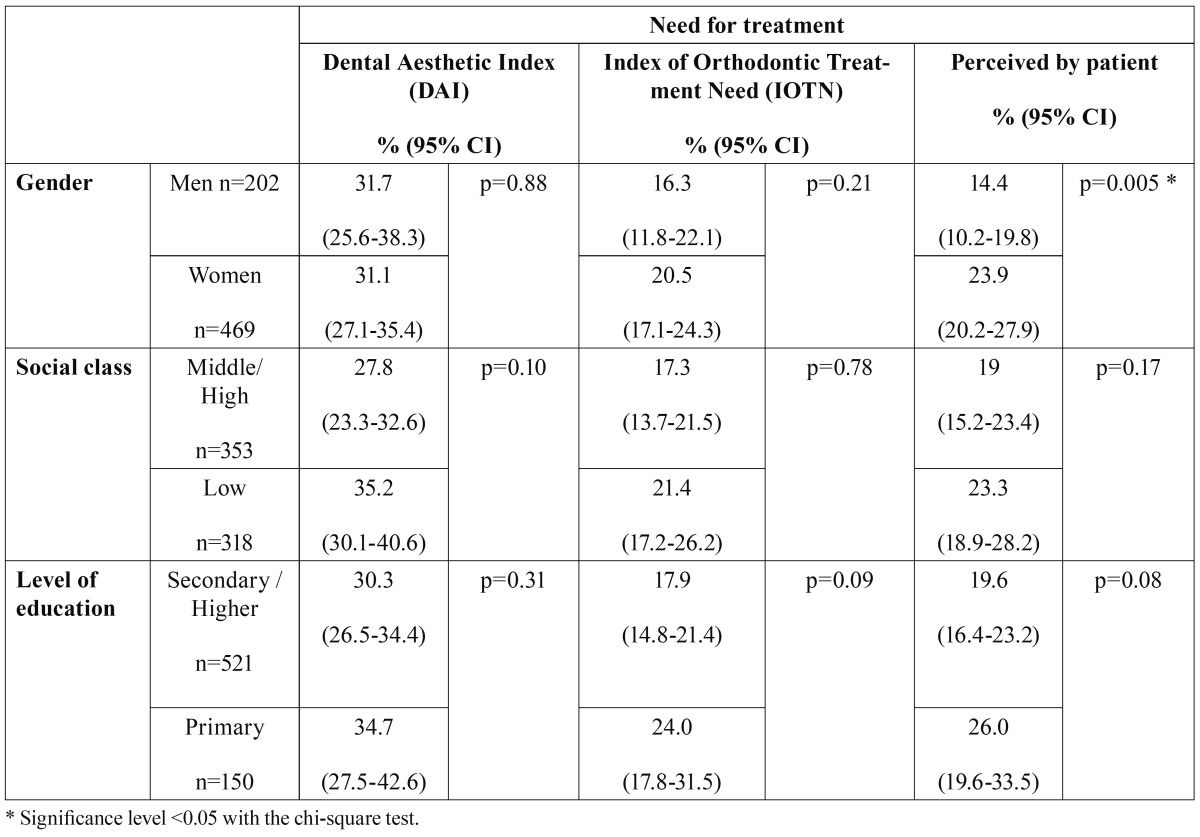
Orthodontic treatment need determined by the DAI and IOTN and perceived by the patient, by gender, social class and level of education.

11.6% of the sample (78 subjects) stated that they had received orthodontic treatment. This had taken place before the patient was 20 years old in 83.3% of these cases, and 73.1% stated that they were satisfied with the treatment they had received. Table 3 presents the previous orthodontic treatment data by gender, social class and level of education. On analyzing the gender distribution of the patients who had received orthodontic treatment, the percentage of men (8.4%) was found to be lower than that of women (13%), although the differences were not significant. However, significant differences were found between ‘middle/high’ (15%) and ‘low’ (9%) social class and between ‘secondary/higher’ (14%) and ‘primary’ (3.3%) education.

92.5% of the sample considered dental esthetics ‘very important’ or ‘quite important’, while 7.5% considered it ‘not very important’ or ‘not at all important’.

## Discussion

Choosing public health centers as the sampling points might be considered controversial. Similar studies of the same age group in Spain have employed the same sampling method. The regional government’s 2005 health survey showed that in the 25-44 year-old population, only 5% are private healthcare users, 72% use the public health system and 23% do not attend any health center. These data suggest that the sample employed in this study is adequately representative.

Another aspect that needs to be considered is the distribution of the sample by gender, since 30.1% of the 671 subjects were men and 69.9% women. This is mainly because of the greater number of women visiting the health centers. The possibility of reducing the number of examinations of women in order to achieve an optimum balance was considered, but it would have reduced the sample size considerably, thus weakening the study.

There is some debate as to whether or not subjects with previous orthodontic treatment should be included in such studies. Some authors exclude them (11-13), while others include them (14-16). In the present study, patients receiving orthodontic treatment at the time of examination were excluded and those who had received orthodontic treatment in the past were included. The reasons were that 83.3% of the former had been treated before they were 20, so evidently an aging process had taken place over the past 15 to 20 years, with the possibility of recurrence, and also that on analyzing the results, no statistically significant differences in treatment need were found between those who had and had not received previous orthodontic treatment.

As regards the indices employed to determine objective orthodontic treatment need, the DAI and the DHC (dental health component) of the IOTN (9) are two of the best-known internationally. A number of researchers (17,18) prefer not to use the IOTN aes-thetic component (AC) because of the difficulty that patients have in identifying with one of the 10 photographs. Many studies have found that the orthodontic treatment need results differ considerably according to whether it is measured objectively through the DHC or measured by the IOTN-AC (8,17,19-22). Some authors (13,21) state that the IOTN DHC can be used as a tool to determine which patients should or should not receive orthodontic treatment, even though these results will not match the need determined by the IOTN AC.

Comparison between the present study and others conducted previously is complicated, as the methods employed are different. Studies in young adult populations show similar results to those obtained in the present case, with objective treatment needs ranging between 20% and 30% (8,12,15,23,24). Onyeaso and cols. (16) and Hassan (21) encountered higher values – treatment needs of 48.8% and 71.6% respectively but their samples present an important selection bias in that they were made up of patients visiting orthodontics units for treatment. Equally, Riedmann and Berg (25) found a 60.2% treatment need, but their sample was composed of 88 orthodontic patients. Tang and So (13) and So and Tang (26), who respectively obtained 54.2% and 53% treatment needs, also display evident sample selection bias.

The low agreement between the two indices employed has been confirmed by other authors such as Johnson and cols. (27), Manzanera and cols. (28) and Hlonga and cols. (29). This is because the DAI and the IOTN DHC are different both in their design and in their content and procedure and because although both attempt to measure need, they do not do so in the same way. The present study also found low agreement between the need determined by the DAI and IOTN and that perceived by the patients.

Profit and Fields consider that the demand for orthodontic treatment is directly related to social class or to income. Others have concluded that persons with higher incomes more frequently demand orthodontic treatment (30). On examining the orthodontic treatment history of the sample by social class and level of education, the present study found statistically significant differences, with a higher proportion of treatment in the middle/high social class and in those with a secondary/higher education.

21% of the sample perceived themselves as needing orthodontic treatment. This perception was significantly higher among women than among men. Similar observations have been reported by Burgersdijk and cols. (30) and Hamamci and cols. (11) and Svedström-Oristo and cols. (17) also found that men were usually more satisfied with their dental esthetics than women.

The treatment need determined by the DAI and IOTN in the present study was slightly higher in the low social class than in the middle/high class, and while these differences are not significant, nonetheless they are indicative of a trend that has been found more strongly in other although it must be remembered that most of these other studies were conducted in a child population.

A global analysis of the relationship between the orthodontic treatment need determined by the indices employed and the patient’s own perception of malocclusion found no clear relationship between objective and perceived need. This observation is in agreement with a number of authors (8,11,13,15). It should not be forgotten that the reasons for deciding to undergo orthodontic treatment involve many different factors, including socioeconomic ones, although most of these factors will depend to a large degree on the person’s psychological state and on personal and cultural values and principles, which are difficult to measure with standard indices.
